# Psychosocial Safety Climate of Employees During the COVID-19 Pandemic in Iran: A Policy Analysis

**DOI:** 10.1017/dmp.2020.370

**Published:** 2020-10-12

**Authors:** Hamed Seddighi, Maureen F. Dollard, Ibrahim Salmani

**Affiliations:** Student Research Committee, University of Social Welfare and Rehabilitation Sciences, Tehran, Iran; PSC Observatory, Centre for Workplace Excellence, Justice and Society, University of South Australia, Adelaide, South Australia, Australia; Department of Health in Disaster and Emergency, School of Public Health, Shahid Sadoughi University of Medical Sciences, Yazd, Iran

**Keywords:** COVID-19, human resources management, Iran, mental health, occupational health, policy analysis, psychosocial safety climate

## Abstract

**Objective:**

Iran is among the top 15 countries in the world in terms of coronavirus disease (COVID-19) infection rates. The numbers of infections and deaths are still increasing in September 2020. This study aims to investigate the impact of the policies on terminating the quarantine period on the perception of psychosocial safety by employees and workers in Iran.

**Methods:**

In this study, policy announcements and regulations, media reports, and the results were collected from 2 previously published population surveys that collected employees’ views of the government approach to quarantine. The information thus collected was then analyzed using the “What is the Problem Represented (WPR)” approach for data analysis introduced by Carol Bacchi, and focuses on the question, “What effects are produced by the representation of the problem?”

**Results:**

The Iranian Government decided to quarantine people and close most sectors during the New Year holidays in Iran in March 2020. The duration of quarantine was only 2 weeks, and the government then ordered government organizations and industrial companies to reopen. The advantage of a short quarantine period is assumed to be the reinstatement of productivity while the disadvantage is the likely risk of further transmission of the virus.

**Conclusion:**

The government approach to and communication about the quarantine period has neglected to consider the psychosocial safety climate of employees, who have to go to their workplaces using buses, subways, or other vehicles, and who are under pressure mentally because of fear of infection, dismissal for non-attendance, and the consequent economic problems. The government approach necessarily impacts on the perceived psychosocial safety climate of employees, and hence influences the causes of work stress. If the psychosocial safety climate is not considered and improved, it may reduce the quality of services and products, and increase accidents.

On February 19, 2020, Iran reported its first confirmed case of coronavirus disease (COVID-19) infection.^1^ Iran is among the top 15 countries in terms of people infected with COVID-19 in the world until September 1, 2020.^2,3^ The number of infected people and deaths is still increasing in September 2020. Iran has confirmed 404 648 coronavirus infection cases of which 94% of them recovered and 6% of infected people died until September 15, 2020.^4^ Figure 1 illustrates the COVID-19 death toll in Iran.

**FIGURE 1:**
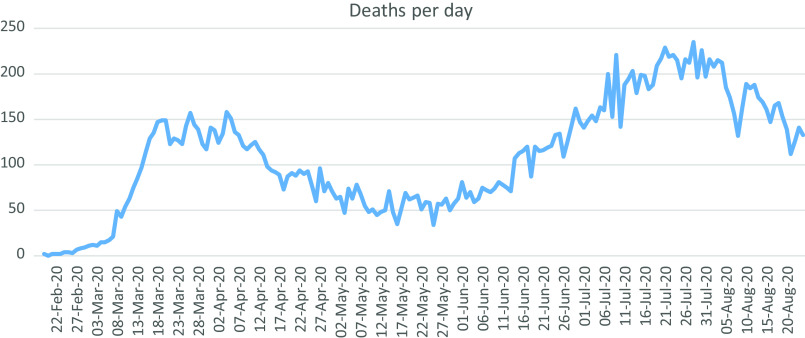
Number of Deaths Per Day Due to COVID-19 in Iran.

A lot has been done so far to survey the effects of the COVID-19 outbreak on the mental health of health workers around the world. However, further research is needed to analyze the effects of the COVID-19 outbreak on the health of employees and workers in other public and private sectors.^5^ A study conducted in Singapore shows that fear of the COVID-19 outbreak and subsequent misconduct have been seen in workers of some sectors, such as transportation and food.^6^ Also, the employees on the deck of a ship felt a great deal of anxiety for fear that the disease would spread from passengers to them.^6^ Fear is an instinctive defense response in humans and animals essential for survival. However, chronic and disproportionate fear can be dangerous to one’s health and may lead to various mental disorders. Pandemics affect the mental health of a large number of people more so than their physical health, and the adverse consequences of such mental disorders in individuals and society may last longer.^7^ For example, at the time of the Ebola outbreak, fear-related behaviors affected people, individually and collectively, and increased mental disorders among them. Now, with the spread of COVID-19, easy access to communication technologies and the transmission of information, or misinformation, can easily increase harmful social reactions, such as anger and aggressive behavior.^7^ A study in China about the effects of the COVID-19 epidemic on mental health indicated that workers, especially migrant workers who more often used public transport to get to work, were more likely to have mental disorders during the COVID-19 outbreak. Fear of infection, fear of being late, and fear of losing a job have been among the stresses of these workers.^8^ Fear of infection and failure of access to preventive materials such as face masks in Hong Kong have damaged workers’ mental health during the COVID-19 pandemic.^9^ A study in India found that various marginalized minorities, including homeless people, daily-wage workers, and migrant workers, were the most likely victims of the COVID-19 pandemic. Fear of the COVID-19 outbreak, unemployment, poverty, and starvation threatens these people at the same time.^10^

The connection between work and mental health and preventing ill health is a global concern seriously emphasized by international organizations, such as the World Health Organization and the International Labor Organization.^11,12^ The term *Psychosocial Safety Climate* (PSC) is used as both a concept and a theory proposed by Dollard and colleagues. The concept refers to shared perceptions about policies, practices, and procedures for the protection of worker psychological health and safety. The theory posits that these shared perceptions are a major influence on worker mental health.^9^ PSC is a theory for employees’ mental health. The theory proposed by Dollard and colleagues refers to shared perceptions regarding “policies, practices, and procedures for the protection of worker psychological health and safety.”^13^

PSC influences the causes of work stress.^14^ Work stressors are defined as: “those aspects of work design and the organization and management of work, and their social and environmental contexts, which have the potential for causing psychological, social, or physical harm.”^15^ Recovery from work stress usually happens in the evening and weekend as time off. Recovery will be incomplete if the employee is exposed to a stressor too intensely or too frequently.^14^ There is confirmed evidence for many consequences of work stress including worker health, quality of services and products, accidents, injuries, conflicts with family and at work, medications, and suicide.^14^ As the end of lockdown approaches in Iran and the public and private sectors are beginning to go back to work on April 11, the media have reported on overcrowded subway wagons.^16^ In Tehran, with a large number of public and private sector workers and a low capacity public transport system, the situation is more complicated than ever before. At a meeting on April 13, the Deputy Minister of Health announced that public transport had been responsible for 26.5% of the COVID-19 outbreak in the country.^17^ Tehran’s Deputy Mayor announced on May 6, 2020, that a health screening test performed on more than 15 500 urban service workers had shown that 2610 people had symptoms similar to COVID-19.^18^ Municipality workers in various cities, including Tehran, have repeatedly protested against the lack of protective equipment, including face masks, gloves, and disinfectants.^19^ In addition, 31 taxi drivers in the country have died from COVID-19 and 450 have been infected.^20^

Bringing the quarantine to an end has its pros and cons. Proponents of bringing the quarantine period to an end refer to the economic problems of the population, while advocates for a longer quarantine period refer to the continued risks of COVID-19 transmission. However, the PSC of people who have to come to work with fear and stress has been neglected in this discourse. This study aims to investigate and analyze the implementation of the Iranian Government’s policy on quarantine for COVID-19 and the impact of the policy on terminating the quarantine on the perception of psychosocial safety of employees and workers in Iran.

## METHODS

In this study, we investigate the impacts of the Iranian Government’s COVID-19 quarantine policies on the PSC of workers and employees. We collected regulations and official authorities’ interviews from February 20 to May 21, 2020. The documents selected included all national quarantine strategy documents, as well as health documents, formal letters, national instructions, governmental orders, and authorities’ interviews with the media (see Table 1). All identified policy documents were available online and obtained through searches of a range of national government websites, including the official website of the Iranian Government, the Ministry of Health (MOH) website, the Ministry of Industry, Mine, and Trade (IMT) website, and the website of Administrative and Recruitment Organization. We also performed Google searches to find related policies using a combination of key terms, including *worker*, *employee*, *mental health*, *job*, *quarantine*, *mental health*, and *COVID-19*. Opinions of national authorities that were reflected in the media were used to identify policy resistance or silence and to offer another view of the quarantine policy.

**TABLE 1:** Policies and Guidelines Selected for the Analysis

Government’s Quarantine Policy	
Ministry of Health (MOH) order to national lockdown	Mar 21
Industry, Mine, and Trade (IMT) Ministry order to open industries	Apr 6
MOH letter in disagreement with early reopening	Apr 7
Iranian President order to open all industries and organizations	Apr 10
Administrative and Recruitment Organization instructions for opening organizations	Apr 11
Iranian Administration System in the COVID-19 pandemic (Iranian Parliament report)	Apr 21
Evaluating the path taken for the COVID-19 pandemic and the requirements of the leading path (Iranian Parliament report)	Mar 7
Media reports that reflected official views on the government’s quarantine policy	February 20 to May 21
**National Surveys**	
National survey to assess the socio-psychological impact of the COVID-19 pandemic in Iran	Apr 16
Iranian people’s views on the prevalence of the COVID-19 pandemic	Mar 14

In order to achieve the aim of this research, 2 research questions (RQ) framed the process of document collection and analysis:

RQ 1: What are the Iranian Government’s policies for bringing the quarantine to an end for private sector workers and public sector employees?
RQ 2: What are the consequences of the Iranian Government’s policy on the PSC of employees?

We have applied “What is the Problem Represented (WPR)” approach for data analysis. The WPR is an approach to policy analysis introduced by Carol Bacchi.^21^ In the WPR approach, 6 questions about the problem are considered, including the problem itself, presupposition for representation of the problem, the problem’s origin, policy silences, effects produced by the representation of the problem, and unproblematic issues.^21^ This study focuses on Bacchi’s fifth question, which asks “What effects are produced by the representation of the problem?” The study assumes 5 phases for a policy wheel, including policy formulation, policy adoption, policy implementation, policy evaluation, and modification.^22^ A policy analysis could address any phase of the policy wheel. This study focuses on the analysis of policy implementation for employees during the novel COVID-19 outbreak in Iran.

## RESULTS

### Implementation of the Government’s Quarantine Policy

**FIGURE 2:**
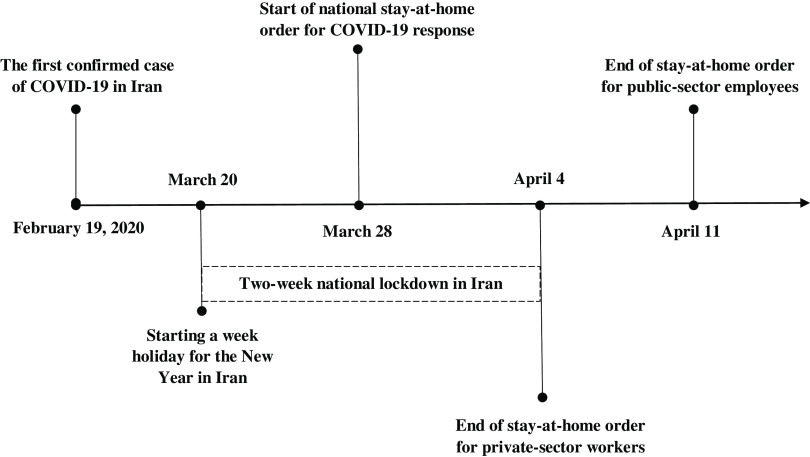
Time Frame of the Iranian National Policy for the Quarantine as a Result of COVID-19.

As Figure 2 illustrates, 1 week after the first positive case was identified on February 19, many public places and events including schools, higher education centers and universities, movie theaters, concert performances, competitions, and national sports league were closed in Tehran and other cities.^2^ However, many businesses in the public and private sectors continued to operate. One month later, on March 21, the Iranian New Year holiday began for a week, and all public and private businesses were closed as in previous years. The Iranian Government decided to start public lockdown during the holidays on March 28.^23^ But 1 week later, just after 2 weeks off, the government ordered the private sector to resume operations and the employees to return to work.^23^

On April 6, the Deputy Minister of Industry, Mine, and Trade (IMT) for Trade Affairs sent a letter to the heads of the IMT offices of the provinces, declaring that “We cannot shut down work and production especially during the New Year called by the Supreme Leader as Year of Production Leap. Therefore, the provincial authorities should prevent any interruption in production units of all industrial, mining, and agricultural sectors.”^24^ Following the letter, the MOH warned the Iranian President about the instructions issued by the Ministry of IMT to reopen jobs and business centers.^24^ According to him, the letter was without prior consultation with other government ministries. The health minister of MOH emphasized: “Although the Interior Minister, other colleagues and I firmly believe in taking prompt action to reduce the adverse effects of COVID-19 epidemic on national economy and developing relevant protocols to reopen economic activities after giving special priorities, we hereby warn that any arbitrary action by any state, non-governmental, cultural, religious, and other organizations not approved by health authorities is like igniting a flame which soon entangles the health system and then the economy of country.*”* Three days later, in response to the letter of the MOH, the President ordered all businesses and offices to comply with health protocols. The Vice President and Head of the Administrative and Recruitment Organization of the country gave explanation in this regard: “We first issued a circular on 4 March which urged that the activities of the departments be carried out according to the situations of that day and our knowledge of the scope and different dimensions of the disease. The Guide to the Second Step in Combating COVID-19 is started with a new regulation about social distancing in office environment. All employees, excluding vulnerable ones, are required to be present at their work offices. We ordered that all health protocols are observed in office environments.”^25^ Some people did not agree with the authorities on returning to the workplace because of health considerations. The Director of the World Trade Center of Iran emphasized the need to continue and intensify the social distancing plan and told the Iranian President, “Will you take them out and endanger their lives?”^26^ The Iranian Parliament report criticized the performance of the administration system in Iran during the COVID-19 pandemic.^27^ It was mentioned in the report that the administration system was inactive and wasn’t preparing for providing remote work for the government employees. As a result, the government asked employees to present at their workplace.^27^ Another report by the Iranian Parliament indicated the government’s response to the COVID-19 pandemic as confusing.^28^ The report said the president’s announcement of an early normalization of the situation was having a negative effect on people’s mental health and physical health.^28^

### National Surveys

The Research Center for Culture, Arts and Communication, affiliated with the Ministry of Culture, conducted a national survey to assess the socio-psychological impact of the COVID-19 pandemic to specify the most serious concern of Iranian people in this period. The survey titled “Investigating Citizens’ Perspectives on COVID-19 pandemic” was conducted nationwide by telephone from April 13 to 16, 2020.^29^ The survey found that 61.1% of citizens are opposed to lifting the coronavirus lockdown and reopening the government departments normally, and 57.4% are opposed to restarting all economic activities by the end of April. When it comes to the reasons for non-observance of self-quarantine, the evidence indicates that 53.1% of the participants are not able to observe self-quarantine because of the nature of their job. Then there are “living conditions and the need to earn money” with a frequency of 25.6 %, “feeling no need to be in self-quarantine” with a frequency of 7.7 %, and “employer’s refusal to shut down the work” with a frequency of 3.9 %, among other reasons.^29^ Another survey on the Iranian people’s views on the prevalence of the COVID-19 pandemic in the national level, with a sample size of 1554 people, was conducted by telephone by the Iranian Student Polling Agency (ISPA) dated March 11–14, 2020.^30^ As many as 75.2% of respondents are concerned that they or their family members might be infected with COVID-19, 5.1% are somewhat worried, and 19.6% are just a little bit worried. The comparative results of the data show that women (about 78%) are more concerned than men (about 73%).^30^

### Media Reports

Many newspapers and official news agencies in Iran covered people’s criticisms of the new policy. A member of the Iranian Parliament has noted the people’s concerns: “We have repeatedly received calls from people who are unhappy with the reopening of offices. On the one hand, they are told that everyone should stay at home and be quarantined, and on the other hand, the offices have been opened. Arriving at work usually calls for taking taxi or using public transport while many bosses and managers do not approve telecommuting and leave requests.”^31^ Indicating the dissatisfaction of employees she declared that the reopening of offices would be in contradiction to the decisions of the COVID-19 headquarters and the statement of officials who asked the people to stay home.^31^

## DISCUSSION

Sometimes, applying maximum effort to solve a problem makes it even worse. Well-intentioned solutions sometimes cause policy resistance. In policy resistance, policies are delayed and weakened by unpredicted reactions of people or nature.^32^ In complex systems such as a country, the cause (policy) and effect (consequences) are often distant in time and space. In our case, the government intended to solve the economic problem of the country using a definite policy. However, the fastest solution is not necessarily the best one. Sir Thomas More, the English lawyer, social philosopher, and author once said, “And it will fall out as in a complication of the disease, that by applying a remedy to one sore, you will provoke another; and that which removes the […] one ill symptom produces others.”^33^ One of the consequences the present study focuses on is the effect of such a policy on the PSC of workers and employees.

Forcing employees to go to their work offices during the COVID-19 pandemic is an enormous stressor without recovery. Recovery cannot be reached when the exposure of employees to the COVID-19 outbreak is too intense and too frequent. Forcing employees to go to their workplaces during the COVID-19 pandemic creates fear which is an enormous stressor, too intense because of the known health risks of COVID-19, and too frequent as the fear is present each day, and hence there is no opportunity for recovery from the stressor. The only point emphasized in the policies set by the president with respect to employees’ health protocols is adopting health measures in the workplace. However, policies (implicitly or formally) do not specify how employees will be present at work. Many employees have to travel to work every day by subway and bus. Due to the economic problems in Iran, many wage-earners, especially in Tehran, the capital, are not able to take a taxi or use their private cars. Following the economic crisis in Iran and after the increase of housing costs, the distance between work and home now is long for many employees. As a result, they have to change several buses and subways to get to work. The use of public transport decreases their perception of the psychological and physical safety climate and leads to enormous stress among employees and their families. Because such stress is present every day, the stress accumulates and the worker has no opportunity for recovery from it. Moreover, there are other stressors for the employee. Fear of dismissal or other workplace issues and, consequently, economic problems all make it impossible for the employee to take care of his or her health by avoiding public transport and refusing to go to work. It was indicated by Wong et al.^34^ that there is a correlation between self-reported stress and a lack of workplace policy in place and a lack of protective equipment supply. Another study indicated that employees are in stressful situations and are worried about the pandemic’s implications in their workplace. It was proposed in the mentioned study that the company heads, employers, business leaders, and HR professionals should come forward to accept the challenge to minimize the impact of the pandemic on the employees and ensure their safety, health, and happiness.^35^ Liu et al.^36^ found in their study that a low PSC increases ill-health presenteeism. Ill-health presenteeism is defined as the phenomenon of attending work with health problems and places employees in an area between full work engagement and absence from work, thus directly affecting productivity.^37^ This phenomenon leads to more accidents and errors by employees and workers.^38,39^ Mansour and Trembly^40^ showed that PSC is related directly and negatively to conflict between the work and family. Dormann et al.^41^ indicated that lower levels of PSC among workers and employees increase psychosocial risk for the development of depression. Becher et al.^42^ showed in their study that employees in low PSC environments were 59% more likely to experience circulatory disease symptoms (including myocardial infarction, angina, stroke, or hypertension) than those in high PSC environments.

A pandemic of this severity may be viewed as a public health disaster and, as with other disasters, is influenced by the key axes of social determinants of vulnerability.^43^ As a result, refugee workers and workers in low-income jobs will be more affected by policies on quarantine and return to work.^44^ The situation has also posed many challenges to refugee workers. Most of these workers use public transport and will be fired if they do not show up because they are paid by the day. So, the dilemma facing them is either unemployment or infection and its fear. For example, migrant workers experience mental disorders such as depression and lower quality of life than local populations, under normal conditions. During the COVID-19 pandemic, the situation could be worsening because of health risks and losing jobs. Salmani et al.^45^ showed in their study that Afghan workers in Iran were under pressure to present at their workplace even with risk of infection. In the case of absenteeism, they had feared losing their jobs and income. Thus, in both cases (presenting at workplace or absenteeism), they had a low level of PSC.^45^

In general, the mental health of people during the COVID-19 pandemic is considered in various academic papers and media.^46-49^ Furthermore, the mental health of health sector’s workers during the COVID-19 pandemic has been mostly and particularly emphasized by researchers, global organizations, and decision makers.^50^ However, it seems that the PSC of workers in various sectors around the world, especially in countries such as Iran, should be considered by governments prior to making the policies. Neglecting such a significant issue will result in irreparable personal, social, and economic damage.

## CONCLUSION

The government approach to and communication about the quarantine period has neglected to consider the mental health and PSC of employees, who have to go to their workplaces using buses, subways, or other vehicles, and who are under pressure mentally because of fear of infection, dismissal for non-attendance, and the consequent economic problems. The government approach necessarily impacts on the perceived PSC of employees, and hence influences the causes of work stress. If the PSC is not considered and improved, it will reduce the quality of services and products, and increase accidents.

In the future or in the next waves of this pandemic, some practices and policies at the national level were proposed as follows:

- Gradual reopening of organizations and factories
- Separate policies for metropolitans
- Identifying and addressing the challenges of employees to attend work
- Proper coordination among decision-making organizations in the country during the COVID-19 response
- Paying attention to the mental health of employees and their families along with other dimensions such as their physical health
- Increasing the capacity of public transport to observe physical distancing
- Exploring how Iranian organizations are recognizing PSC through relevant policies, practices, and procedures to protect workers’ mental health for both normal and crisis situations.
- Working with relevant employee representative groups (unions) to ensure that workers’ voice asking for work changes due to COVID-19 is properly addressed.
